# *miR-27b* promotes type II collagen expression by targetting peroxisome proliferator-activated receptor-γ2 during rat articular chondrocyte differentiation

**DOI:** 10.1042/BSR20171109

**Published:** 2018-01-10

**Authors:** Jinying Xu, Shuang Lv, Yi Hou, Kan Xu, Dongjie Sun, Yangyang Zheng, Zechuan Zhang, Xianglan Li, Yulin Li, Guangfan Chi

**Affiliations:** 1 The Key Laboratory of Pathobiology, Ministry of Education, College of Basic Medical Sciences, Jilin University, Changchun 130000, People’s Republic of China; 2 Department of Regeneration Medicine, School of Pharmaceutical Science of Jilin University, Changchun 130000, People’s Republic of China; 3 Department of Neurosurgery, The First Hospital of Jilin University, Changchun 130000, People’s Republic of China; 4 Department of Clinical Medicine, Clinical Medicine College, Jilin University, Changchun 130000, People’s Republic of China; 5 Department of Dermatology, China-Japan Union Hospital, Jilin University, Changchun 130000, People’s Republic of China

**Keywords:** Chondrocyte, Col2a1, Col10a1, Hypertrophy, miR-27b, Pparγ2

## Abstract

MicroRNAs (miRNAs) play an essential role in articular cartilage development and growth. However, the exact mechanisms involved in this process remain unknown. In the present study, we investigated the biological functions of *miR-27b* during hypertrophic differentiation of rat articular chondrocytes. Based on *in situ* hybridization and immunohistochemistry, we report that *miR-27b* expression is reduced in the hypertrophic zone of articular cartilage, but expression of peroxisome proliferator-activated receptor γ (Pparγ) is increased. Dual-luciferase reporter gene assay and Western blot analysis demonstrated that Pparγ2 is a target of *miR-27b*. Overexpression of *miR-27b* inhibited expression of Pparγ2, as well as type X collagen (Col10a1) and matrix metalloproteinase 13 (Mmp13), while significantly promoting the expression of Sex-determining Region-box 9 (Sox9) and type II collagen (Col2a1) at both the mRNA and protein levels. Rosiglitazone, a Pparγ agonist, suppressed Col2a1 expression, while promoting expression of runt-related transcription factor 2 (Runx2) and Col10a1 in a concentration-dependent manner. siRNA-mediated knockdown of Pparγ2 caused an increase in protein levels of Col2a1. The present study demonstrates that *miR-27b* regulates chondrocyte hypertrophy in part by targetting Pparγ2, and that *miR-27b* may have important therapeutic implications in cartilage diseases.

## Introduction

In developing long bones, chondrocytes of the articular cartilage and growth plate show a columnar arrangement, consisting of three characteristic layers with different cellular morphologies and proliferation properties: resting, proliferative, and hypertrophic. Resting and proliferating chondrocytes consist of immature chondrocytes, which have a characteristic shape and secrete a matrix rich in type II collagen (Col2a1) and the proteoglycan aggrecan. As skeletogenesis proceeds, chondrocytes close to the middle of the cartilage undergo further processing, exiting the cell cycle, maturing, and differentiating into hypertrophic chondrocytes. Chondrocyte hypertrophy is a key step during bone development, whereby chondrocytes sharply alter their program and start synthesizing type X collagen (Col10a1), as well as other mineralizing cartilage matrix components. This switch is particularly important for the genesis of osteoclasts and for recruiting blood vessels to the ossification center. Along with invading vascular tissue, osteoblasts appear and synthesize bone matrix, which is laid on and replaces the degraded cartilage scaffold [1–3]. Chondrocyte hypertrophy plays a key role in the synthesis and degradation of cartilage, as well as in bone formation and development through volume enlargement, secretion of special products, and progression to subsequent stages of osteogenesis. Dysregulation of this process has been associated with several disorders, including osteoarthritis and retarded growth, characterized by the imbalance between creation and destruction of cartilage matrix [4,5].

Accumulating evidence suggests that chondrocyte differentiation genes are also regulated by post-transcriptional mechanisms, most significantly by temporally expressed miRNAs [6]. miRNAs are endogenous noncoding RNAs with a length of approximately 22 nts. They participate in multiple biological pathways and play important regulatory roles in animals and plants by targetting mRNAs for degradation or translational repression in a sequence-dependent manner [7]. Dicer is an essential enzyme for the generation of mature miRNAs. Its deficiency in chondrocytes has been shown to cause decreased proliferation and accelerated hypertrophy. Dicer-null mice display growth retardation and growth defects of the maxilla, indicating the importance of dicer-dependent miRNAs in skeletal development [8]. Changes in miRNA expression patterns have also been observed during endochondral ossification, suggesting that miRNAs are likely to play important roles in proliferation, differentiation, and even homeostasis of the entire articular cartilage [9,10]. *miR-1* has been shown to inhibit histone deacetylase 4 (HDAC4) production while increasing expression of runt-related transcription factor 2 (Runx2), Col10a1, and Indian hedgehog through the parathyroid hormone (PTH)-related peptide (PTHrP)-HDAC4 pathway, thus further promoting chondrocyte hypertrophy [11]. *miR-140* is abundantly and relatively specifically expressed in chondrocytes. It has been shown to interact with the PTHrP-HDAC4 pathway indirectly by regulating the expression of myocyte enhancer factor 2C to control chondrocyte differentiation [12].

The transcription factor peroxisome proliferator-activated receptor (Ppar) belongs to the family of ligand-activated nuclear receptors and plays a key role in adipocyte differentiation and glucose homeostasis [13]. Pparγ2, one of the four isomers of Pparγ, is expressed specifically in growth plate chondrocytes, and is regulated by P38 and glycogen synthase kinase 3. Activation of Pparγ promotes adipogenic transdifferentiation of growth plate chondrocytes, while attenuating chondrogenic differentiation. Monemdjou et al. [14] observed that cartilage-specific Pparγ-knockout (KO) mice presented abnormal cartilage growth and development of endochondral ossification [15,16]. These results underscore the important roles of Pparγ in cartilage differentiation.

*miR-27b* has been reported to play a role in a variety of processes, such as tumorigenesis, inflammatory diseases, and adipocyte differentiation, by targetting Pparγ [17–19]. In the present study, given that the distribution of *miR-27b* was previously shown to contrast with that of its target gene, *Pparγ* [20], we investigated the requirement for *miR-27b* during chondrocyte differentiation. Specifically, we attempted to determine whether *miR-27b* repressed chondrocyte hypertrophy and differentiation by targetting Pparγ.

## Results

### Harvesting and identification of resting/proliferative and hypertrophic chondrocytes

**Figure 1:**
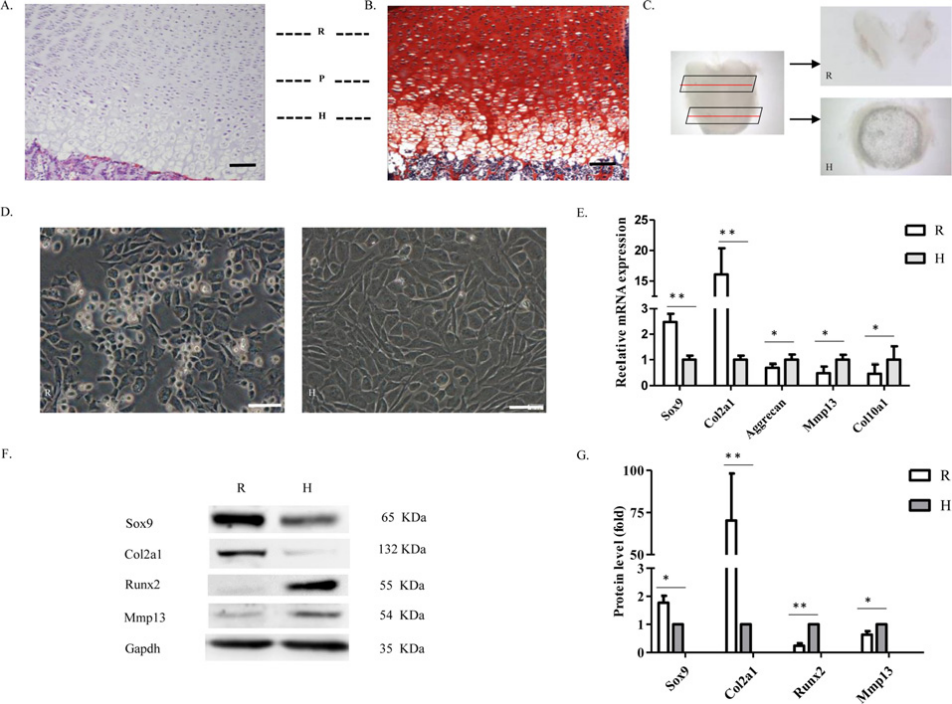
Isolation, cultivation, and identification of resting and hypertrophic chondrocytes (**A**) Hematoxylin and Eosin and (**B**) Safranin O staining of a representative section of newborn rat articular cartilage, indicating the resting (R), proliferative (P), and hypertrophic (H) zones. Bars: 20 µm. (**C**) Stereomicroscope image showing the surface and deep part of a whole articular cartilage specimen isolated in this experiment. (**D**) Cartilage pieces were digested and cultured separately. Morphology of different chondrocytes is shown. Bars: 20 µm. (**E**) qRT-PCR analysis of chondrocyte marker genes: Sox9, Col2a1, aggrecan, and hypertrophy marker genes, Mmp13 and Col10a1. β-actin was used as a reference gene (*n*=3, *P<0.05, **P<0.01). (**F**,**G**) Western blot performed on isolated chondrocytes showing the relevant protein expression of Sox9, Col2a1, Runx2, and Mmp13. Each column represents mean ± S.D. from three independent experiments. Gapdh was used as a loading control (*n*=3, *P<0.05, **P<0.01).

During embryonic and post-natal development, articular cartilage consists of various distinctly differentiated chondrocytes. Hematoxylin and Eosin (Figure 1A) and Safranin O (Figure 1B) staining of femoral articular cartilage sections from newborn rats identified resting, proliferative, and hypertrophic zones on the articular cartilage. Morphologically, articular cartilage chondrocytes show a columnar arrangement from the surface to the deep part of the articular cartilage, where cell volume increases gradually. To better characterize resting/proliferative and hypertrophic chondrocytes, we used a stereomicroscope to isolate the cartilage from resting and hypertrophic zones of articular cartilage (Figure 1C) and then digested them into single cells. After several days of culture, we harvested resting and hypertrophic chondrocytes. The resting cells had smaller volume and clearer margins than the hypertrophic cells (Figure 1D). Real-time reverse transcription-polymerase chain reaction (qRT-PCR) and Western blot showed the expression of chondrocyte marker genes and relevant proteins (Figure 1E–G). As expected, resting chondrocytes expressed Col2a1 and Sex-determining Region-box 9 (Sox9) at high levels, but hardly showed any expression of Runx2 or matrix metalloproteinase 13 (Mmp13), two hypertrophic differentiation markers. Thus, we had successfully mechanically dissected resting and hypertrophic regions individually from post-natal rat articular cartilage.

### Expression of *miR-27b* and Pparγ in articular cartilage

**Figure 2:**
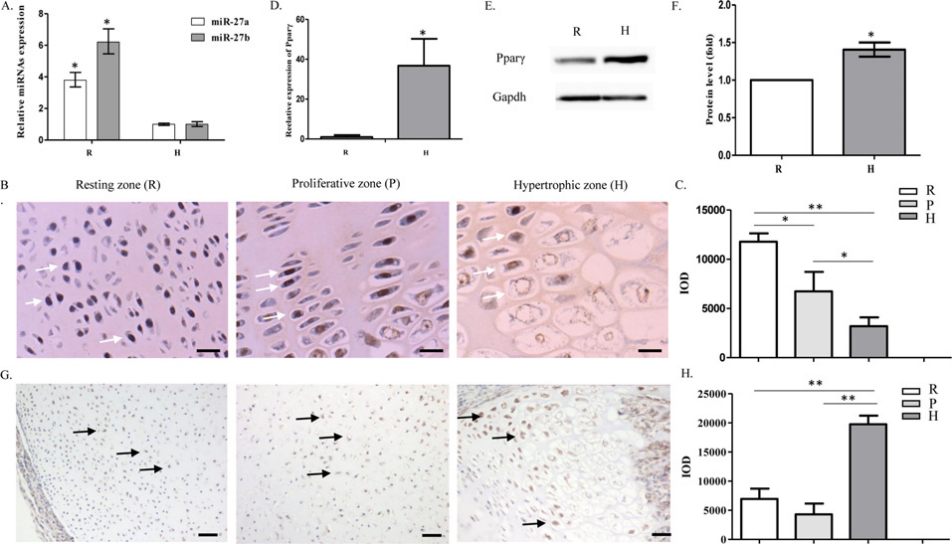
Expression of *miR-27b* is reduced during hypertrophic differentiation of chondrocytes in articular cartilage (**A**) Relative expression of *miR-27a* and *miR-27b* in resting chondrocytes (R) compared with hypertrophic chondrocytes (H). *U6* was used for normalization of qRT-PCR data (*n*=3, *P<0.05). (**B**,**C**) *miR-27b* (white arrows) expression in the resting, proliferative, and hypertrophic zones was detected by *in situ* hybridization in histological sections of articular cartilage. Bar: 25 µm. The graph indicates quantitation of the *in situ* hybridization (*n*=5, *P<0.05, **P<0.01). (**D**) qRT-PCR analysis of *Pparγ* mRNA expression in the resting zone (R) compared with hypertrophic zone (H). *β-actin* was used as a reference gene (*n*=3, *P<0.05). (**E**,**F**) Western blot showing the expression of Pparγ in resting (R) and hypertrophic (H) chondrocytes. Semi-quantitation of Western blots indicating significantly increased production of Pparγ in hypertrophic chondrocytes (*n*=3, *P<0.05). (**G**,**H**) Immunohistochemical staining showing Pparγ expression and location (black arrows) in the resting, proliferative, and hypertrophic zones of articular cartilage. Bar: 50 µm. The graph indicates quantitation of the immunohistochemical staining (*n*=5, **P<0.01).

*miR-27a* and *miR-27b* are highly homologous miRNAs. qRT-PCR showed that *miR-27b* was abundantly expressed in resting chondrocytes and that expression levels decreased significantly when cells differentiated into hypertrophic chondrocytes. The difference was more apparent for *miR-27b* than for *miR-27a* (Figure 2A). In line with the qRT-PCR results, *in situ* hybridization showed how expression patterns changed during differentiation. Simultaneously, it also confirmed the nuclear localization of *miR-27b* and the reduced expression of *miR-27b* during hypertrophic chondrocyte differentiation (Figure 2B,C). Next, we analyzed the expression and location of Pparγ in different regions of articular cartilage. qRT-PCR and Western blot results indicated up-regulation of Pparγ in hypertrophic chondrocytes compared with resting chondrocytes (Figure 2D–F). Immunohistochemical staining showed that Pparγ was highly expressed in the hypertrophic zones (Figure 2G,H).

### Pparγ2 is a target of repression by *miR-27b*

**Figure 3:**
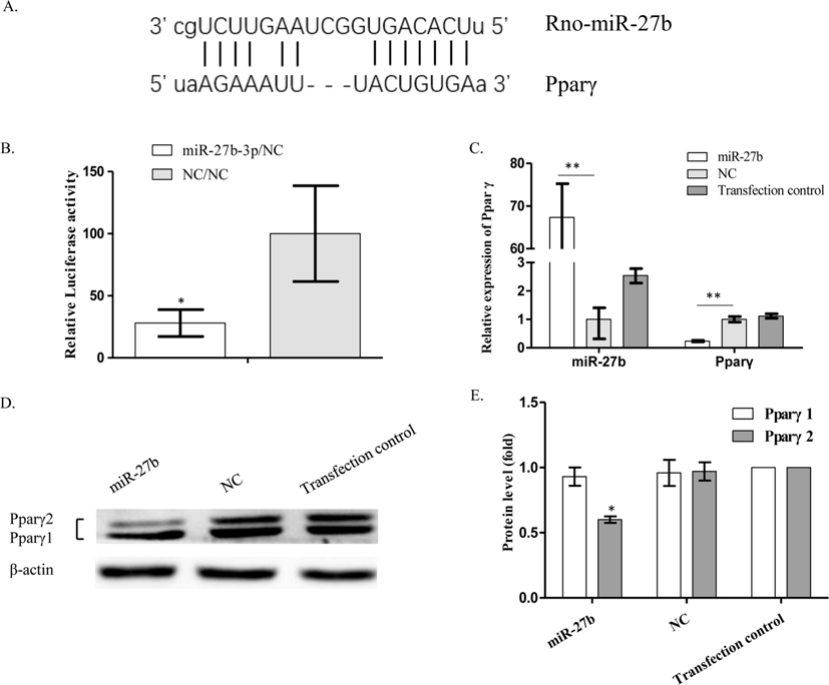
Pparγ2 is a target gene of *miR-27b* (**A**) The predicted binding sites on the 3′-UTR of Pparγ and *miR-27b* are indicated (microrna.org). (**B**) Interaction of *miR-27b* with intact putative *miR-27b*-binding sites on the 3′-UTR of Pparγ. Fold-change ± S.E.M. value of *Renilla* luciferase activity in NC mimic-transfected cells compared with *miR-27b* mimic-transfected cells (*n*=3, *P<0.05). (**C**) qRT-PCR showing the relative expression of *Pparγ* after transfection with *miR-27b* mimics compared with NC-transfected cells. β-actin was used as a reference gene (*n*=3, **P<0.01). (**D**) Western blot performed on transfected cells demonstrating the expression of Pparγ protein. β-actin was used as a loading control. (**E**) Semi-quantitation of Western blots showing the fold-change of Pparγ1 and Pparγ2 protein expression (*n*=3, *P<0.05).

Bioinformatics analysis suggested that *Pparγ* was a potential target gene of *miR-27b*. In addition, miRBase showed that the binding sites of *miR-27b* were evolutionarily conserved in both humans and rats (Figure 3A). Interactions of *miR-27b* with complementary sequences in the 3′-UTR of predicted target genes were analyzed using luciferase reporter assays. Hypertrophic chondrocytes were transfected with *miR-27b* mimics or negative control (NC) mimics and then cotransfected with a luciferase reporter construct containing the intact putative *miR-27b*-binding sequence of the 3′-UTR of Pparγ. Luciferase activity was significantly reduced in the presence of *miR-27b* mimics (Figure 3B). qRT-PCR demonstrated a significant decrease in *Pparγ* mRNA expression in mimic-transfected cells compared with the NC mimic or transfection reagent groups (Figure 3C). Intriguingly, the result was confirmed at the protein level by Western blot, but only for Pparγ2 and not for Pparγ1, another isoform of Pparγ (Figure 3D,E). These findings suggest that Pparγ2 is a target of *miR-27b*, and that expression of Pparγ protein is regulated by *miR-27b* via mRNA degradation and translation inhibition.

### *miR-27b* promotes expression of Col2a1 and inhibits chondrocyte hypertrophy

*miR-27b* expression was lower in hypertrophic chondrocytes than in resting chondrocytes. We thus investigated whether, at higher levels, *miR-27b* inhibited hypertrophic chondrocyte differentiation.

**Figure 4:**
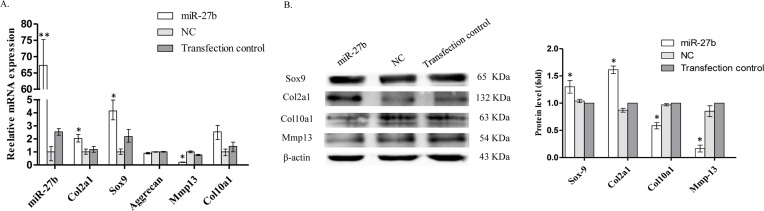
*miR-27b* promotes expression of Col2a1 and inhibits chondrocyte hypertrophy (**A**) qRT-PCR shows the relative expression of relevant genes after transfection with *miR-27b* mimics compared with NC mimics. *U6* was used as a reference gene when detecting *miR-27b* expression and *β-actin* was used as a reference when detecting expression of *Col2a1, Sox9, aggrecan, Mmp13*, and *Col10a1* (*n*=3, *P<0.05, **P<0.01). (**B**) Western blot performed on transfected cells demonstrating the expression of relevant proteins. β-actin was used as a loading control. Semi-quantitation of Western blots showing the fold-change in protein expression (*n*=3, *P<0.05, **P<0.01).

Changes in the relative expression of relevant genes were assessed by qRT-PCR following transfection with *miR-27b* and NC mimics. In the *miR-27b* mimic group, expression of *Col2a1* and *Sox9* mRNA was significantly increased, that of Mmp13 declined significantly, whereas that of Col10a1 and aggrecan did not change significantly (Figure 4A). Western blot results confirmed increased protein expression of Col2a1 and Sox9, but revealed decreased expression of both Col10a1 and Mmp13 (Figure 4B). These findings indicate that *miR-27b* played a crucial role in maintaining chondrocytes in an undifferentiated status, while simultaneously inhibiting hypertrophic differentiation.

### *miR-27b* and siRNA-Pparγ inhibit the production of lipid droplets in hypertrophy-enhancing medium

**Figure 5:**
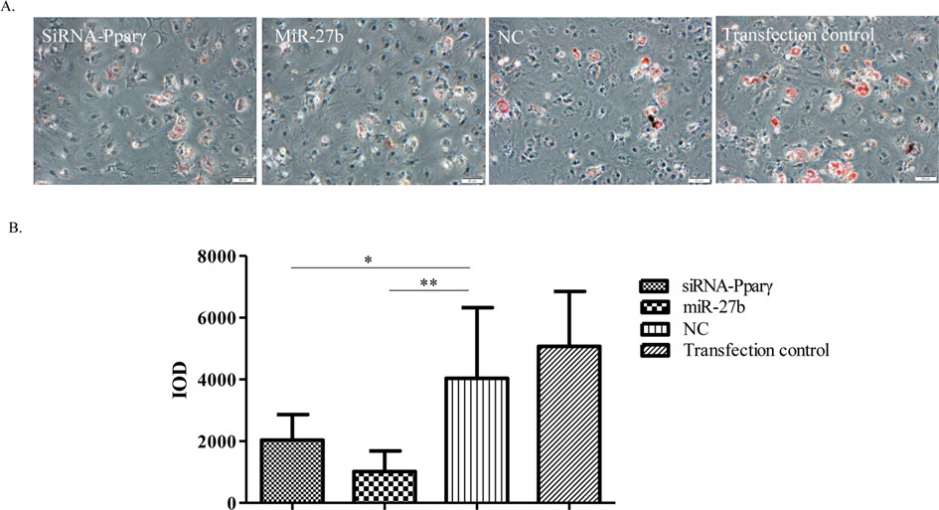
*miR-27b* and siRNA-Pparγ inhibit the production of lipid droplets in hypertrophy-enhancing medium (**A**) Hypertrophic chondrocytes were treated with siRNA-Pparγ, *miR-27b* mimicNC-mimic, and transfection control, respectively. Seven days after induction of hypertrophy in differentiation medium, chondrocytes became adipocytes. Lipid droplets (red) in chondrocytes are revealed by Oil Red O staining. Bar: 50 µm. (**B**) The integrated optical density (IOD) of lipid droplets decreased significantly in *miR-27b* mimic-transfected cells and siRNA-Pparγ-transfected cells compared with the NC and transfection control group of cells (*n*=5, *P<0.05, **P<0.01).

Pparγ is the master regulator of adipocyte biology. To further explore the relationship between *miR-27b* and Pparγ2 during hypertrophy differentiation, we knocked down Pparγ2 with siRNA (siRNA-Pparγ), and treated the siRNA-Pparγ transfected cells, *miR-27b* mimic-transfected cells, NC mimic-transfected cells, and transfection control group cells with hypertrophy-enhancing medium, respectively. Accordingly, a subpopulation of chondrocytes showed the potential to transdifferentiate into adipocytes. Oil Red O staining revealed lipid droplets in some chondrocytes, with *miR-27b* mimic-transfected cells and siRNA-Pparγ transfected cells showing significantly lower staining compared with the other two groups (Figure 5A,B). This finding indicates that *miR-27b* may play key roles in suppressing adipocytic differentiation of chondrocytes and possibly influencing the hypertrophic differentiation process by repressing Pparγ2 translation.

### Effect of rosiglitazone and siRNA-Pparγ on the differentiation of chondrocytes

#### Overexpression of Pparγ inhibits the expression of Col2a1 and promotes hypertrophic differentiation in resting chondrocytes

**Figure 6:**
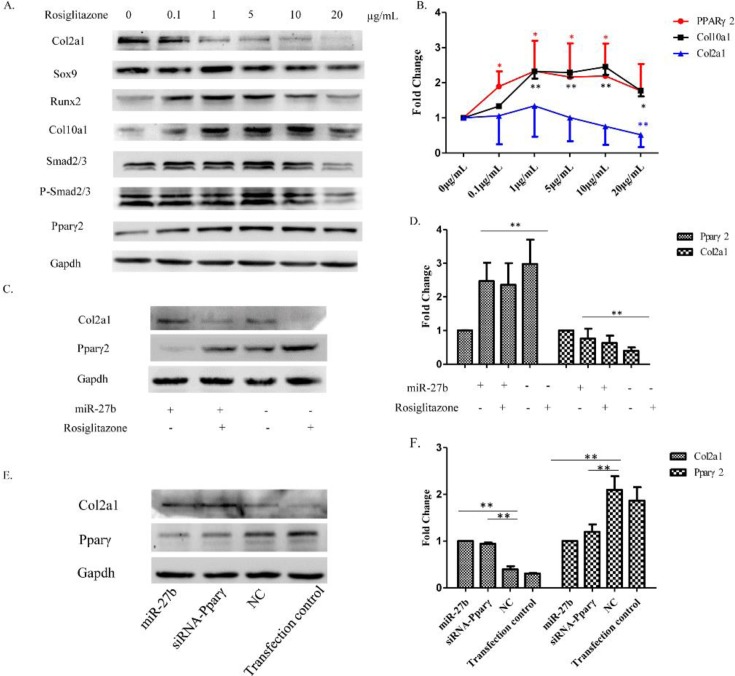
Effect of overexpression or knockdown of Pparγ on the differentiation of chondrocytes (**A**) Resting chondrocytes plated in a monolayer were treated with increasing concentrations of rosiglitazone (0–20 µg/ml). Western blot showing the expression of selected proteins. Gapdh was used as a loading control. (**B**) Fold-change of Pparγ, Col10a1, and Col2a1 at different concentrations of rosiglitazone compared with untreated resting chondrocytes (*n*=3, *P<0.05, **P<0.01). (**C**) Resting chondrocytes were concomitantly treated with *miR-27b* mimics (75 nM) and rosiglitazone (5 µg/ml). Western blot showing the expression of selected proteins. (**D**) Semi-quantitation of Western blots showing the fold-change of Col2a1 and Pparγ2 protein expression. Gapdh was used as a loading control (*n*=3, *P<0.05, **P<0.01). (**E**) Hypertrophic chondrocytes were transfected with *miR-27b* mimics (75 nM) and siRNA-Pparγ (50 nM), respectively. Western blot showing the protein level of Col2a1 and Pparγ. (**F**) Semi-quantitation of Western blots showing the fold-change of Col2a1 and Pparγ2 protein expression. Gapdh was used as a loading control (*n*=3, *P<0.05, **P<0.01).

Rosiglitazone is an agonist of Pparγ. To investigate the biological function of Pparγ2 in chondrocytes, we artificially enhanced Pparγ2 levels by treating resting zone chondrocytes with various concentrations of rosiglitazone (0–20 µg/ml). Western blot results showed a concentration-dependent response of various proteins to rosiglitazone (Figure 6A). Pparγ2 up-regulation was detected with 0–1.0 µg/ml of rosiglitazone and a stable plateau was reached with 1.0–10 µg/ml. At higher concentrations of rosiglitazone (20 μg/ml), the Pparγ2 level was significantly decreased. The same expression pattern was also observed for Runx2 and Col10a1. Finally, Col2a1 expression was repressed already at 1 µg/ml rosiglitazone (Figure 6B). These findings indicate that Pparγ2 could stimulate hypertrophic chondrocyte differentiation and inhibit Col2a1.

#### miR-27b promotes expression of Col2a1 by targetting Pparγ2 translation

After discovering that *miR-27b* could promote the expression of Col2a1, we investigated whether this function of *miR-27b* depended on repression of Pparγ2 expression. To test this hypothesis, we treated chondrocytes simultaneously with 20 μg/ml rosiglitazone and *miR-27b* or NC mimics. Western blot demonstrated that rosiglitazone significantly inhibited the up-regulation of Col2a1 induced by *miR-27b* mimics (Figure 6C,D). Accordingly, *miR-27b* promoted the expression of Col2a1 by inhibiting the post-translational processing of Pparγ2.

#### Knockdown of Pparγ promotes expression of Col2a1 in hypertrophic chondrocytes

To further investigate the function of Pparγ2 in the differentiation of chondrocytes, we detected the Col2a1 protein levels in *miR-27b* mimic-transfected cells, siRNA-Pparγ transfected cells, NC mimic-transfected cells, and transfection control group cells. Western blot demonstrated that both *miR-27b* mimic-transfected and siRNA-Pparγ transfected cells could significantly promote hypertrophic chondrocytes to produce Col2a1 compared with NC mimic transfected cells or transfection control group (Figure 6E,F). These findings suggest that knockdown of Pparγ2 or treatment with *miR-27b* mimic acts similarly in the regulation of Col2a1 expression.

## Discussion

In the present study, we report for the first time that *miR-27b* is significantly down-regulated during hypertrophic cartilage differentiation, and that overexpression of *miR-27b* promotes the expression of Col2a1 and Sox9. Furthermore, we confirm that Pparγ2 is a target gene of *miR-27b*, which controls chondrocyte differentiation by regulating Pparγ2 protein expression.

In articular cartilage, gene expression varies with the different stages of chondrocyte differentiation. Immature chondrocytes of the resting zone express the transcription factors Sox5, Sox6, and Sox9, and secrete a matrix rich in Col2a1 and the proteoglycan aggrecan. Early hypertrophic chondrocytes express PTH 1 receptor, Indian hedgehog, and Col10a1, whereas expression of Sox5, Sox6, and Sox9 gradually decreases. At the final stage of chondrocyte hypertrophy, cells mainly express Runx2, Col10a1, Mmp13, vascular endothelial growth factor A, and osteopontin. These different gene expression patterns are thought to be strictly regulated by multiple players, such as growth factors, cytokines, and related signaling pathways, but the underlying mechanism regulating chondrocyte differentiation has not been fully elucidated. It has been estimated that as many as 60% of coding genes are regulated by miRNAs [21]. As with other organs or tissues, it is likely that some miRNAs are involved in modulating chondrocyte differentiation and play pivotal functions through post-transcriptional control of target genes. To date, an increasing number of miRNAs has been shown to play crucial roles in chondrocyte differentiation of developing cartilage, including *miR-140* [22,23], *miR-199* [24], *miR-221* [25], *miR-1* [11], *miR-381* [26], and *miR-455-3P* [27]. McAlinden et al. [28] used laser capture microdissection to separate the three chondrocyte populations from distinct regions of developing human cartilage, and determined miRNA expression profiles. Expression of 45 analyzed miRNAs within the precursor chondrocyte region was at least two-fold higher than that in hypertrophic cells. Specifically, and in line with our data, *miR-27a* and *miR-27b* expressions were increased by 2.96-fold and 2.32-fold, respectively. In the present study, we show that after treatment with *miR-27b*, Sox9 and Col2a1 expression was significantly up-regulated at both mRNA and protein levels, whereas Mmp13 expression declined. These results imply that *miR-27* is essential for maintaining the undifferentiated phenotype of resting chondrocytes and that decreased expression of *miR-27b* further stimulates hypertrophic chondrocyte differentiation.

Similar to other miRNAs, *miR-27b* exerts a biological function in chondrocytes by acting on its target genes, such as Pparγ [29], myocyte enhancer factor 2C [30], Sp1 transcription factor [31], and Mmp13 [32]. Here, we focussed on Pparγ as a major target gene because of its role in regulating Col2a1. Increasing evidence suggests that Pparγ plays important roles during chondrocyte differentiation [14]. However, the function of Pparγ in chondrocytes remains controversial. Pparγ-overexpressing chondrocytes showed decreased expression of the chondrogenic genes Col2a1 and aggrecan [16]. During *in vitro* cultivation of chondrocytes from newborn mouse xiphoids, the RNA level of lipoprotein lipase, an adipocyte-specific gene, increased steadily, whereas that of Col2a1 decreased [33]. However, in cartilage-specific Pparγ-Knockout mice, the expression of Sox9 and Col2a1 appeared to be significantly reduced [14]. In contrast, we found that in rat articular cartilage, expression of Pparγ was inversely proportional to that of Col2a1. Moreover, after treatment of chondrocytes with rosiglitazone, a Pparγ agonist, Col2a1 and Sox9 levels decreased in a concentration-dependent manner. Pparγ exists as two isoforms, Pparγ1 and Pparγ2. Both proteins are products of the same gene, but the gene expression is regulated by different promoters. Pparγ2 has additional amino acids at the NH_2_-terminal end and has a distinctly different protein structure from Pparγ1 [15]. Pparγ2 is known to be expressed in growth plate chondrocytes and to be involved in lipid metabolism; however, the biological roles of Pparγ2 during chondrocyte differentiation are still not clear. We suggest that Pparγ, especially, Pparγ2 is essential for normal chondrocyte differentiation, and that optimal expression of Pparγ is crucial for ensuring its biological function. Excessive overactivation or inactivation of Pparγ may have different effects on chondrocyte differentiation.

In chondrocytes, the transforming growth factor β (TGF-β)-Smad2/3 pathway is involved in regulating Col2a1 expression. Pparγ has been shown to inhibit TGF-β-Smad2/3 [34,35]. We show that, after treatment of chondrocytes with rosiglitazone, Smad2 and Smad3 protein levels were up-regulated in a Pparγ concentration-dependent manner; however, the levels of the phosphorylated forms of Smad2 and Smad3 remained unaltered. These results indicate that at least in rat chondrocytes, Pparγ regulates Col2a1 expression independently of the TGF-β-Smad2/3 pathway. Mmp13 is a typical hallmark of hypertrophic chondrocyte differentiation and a target gene of *miR-27b* [32]. We report that after artificial enhancement of *miR-27b* levels in rat hypertrophic chondrocytes, both *Mmp13* mRNA and protein levels were significantly down-regulated, confirming previous studies suggesting that Mmp13 is a target of *miR-27b* [32]. Mmp13 is a major member of the Mmp family, which is involved in breaking down extracellular matrix components, such as collagen and aggrecan. Recently, Li et al. [36] reported that in human spine nucleus pulposus tissue, chondrocyte Col2a1 expression was positively regulated by *miR-27b* and that this function was attenuated by artificial overexpression of Mmp13. Based on these results, we speculate that *miR-27b* promotes Col2a1 expression in rat articular chondrocytes by down-regulating Pparγ2 and by repressing Mmp13 protein expression.

In conclusion, in rat articular chondrocytes, up-regulation of *miR-27b* promotes expression of Sox9 and Col2a1 by targetting Pparγ2, and influences hypertrophic differentiation by suppressing Mmp13 expression. Although the function of *miR-27* in chondrocytes remains only partially understood, these findings provide new insights for exploring the mechanism of chondrocyte differentiation during the development of articular cartilage. Furthermore, these findings suggest that up-regulating *miR-27b* or preventing *miR-27b* expression in articular cartilage could be an effective therapeutic or preventive treatment against chondrocyte-related abnormalities and degenerative joint diseases such as osteoarthritis.

## Materials and methods

### Animals

All animals were supplied by the Experimental Animal Center of Jilin University. All procedures involving animals were approved by the Ethics Committee of Jilin University and conformed to regulatory standards. The ethical approval code was 2017-120.

### Harvesting of cartilage tissue and primary chondrocyte culture

Fifteen newborn Wistar rats (1–3 days) were killed by CO_2_ suffocation followed by immersion in 75% ethanol for 5 min for disinfection of the entire body. Whole knee articular cartilage was obtained; the bone was excluded to avoid excessive connective tissue. Each block of cartilage was divided into three pieces and cartilage from resting and hypertrophic zones was harvested for subsequent experiments. Cartilage was washed twice with PBS and cut into pieces of 1 × 1 × 1 mm^3^. The pieces were digested with 0.15% type II collagenase in Dulbecco’s modified Eagle’s medium (DMEM)/F12 (1:1) supplemented with 5% (v/v) FBS at 37°C for 12–16 h. The digest was passed through a 40-μm diameter filter screen and centrifuged. Chondrocytes were collected and resuspended in DMEM/F12 (1:1) with 10% FBS and seeded on to culture dishes. Cultures were maintained in a humidified atmosphere of 5% CO_2_ at 37°C. Once cells had reached more than 90% confluence (passage 0), they were passaged using 0.25% trypsin/EDTA. Experiments were performed using cells after three passages.

### qRT-PCR assay

Total RNA was extracted using the miRNeasy Mini kit (Qiagen, Germany), and cDNA was synthesized using the GoScript Reverse Transcription System (Promega, U.S.A.) for qRT-PCR, following the manufacturer’s instructions. Briefly, the reaction was performed in a final volume of 20 µl with 500 ng of total RNA, 1 µl of oligo(dT)^15^, 4 µl of GoScript 5× reaction buffer, 4 µl of MgCl_2_, 1 µl of PCR nucleotide mix, 0.5 µl of recombinant RNasin ribonuclease inhibitor, 1 µl of GoScript reverse transcriptase, and nuclease-free water to complete the final volume, and then incubated at 25°C for 5 min, 42°C for 55 min, and 70°C for 15 min.

For detection of miRNAs, 1000 ng of total RNA per sample was used with the All-in-One miRNA qRT-PCR Detection Kit (GeneCopoeia, U.S.A.). The reaction was performed in a final volume of 25 µl with 5 µl of 5× PAP/RT buffer, 1 µl of 2.5 U/µl Poly A Polymerase, 1 µl of RTase Mix, and RNase-free water to complete the final volume. The mixture was incubated at 37°C for 60 min and 85°C for 5 min.

Mature *miR-27* (*miR-27a* and *miR-27b*) expression levels were measured with the All-in-One miRNA qRT-PCR Detection Kit (GeneCopoeia). U6 was used as a reference gene to normalize the relative amount of miRNA, and samples were analyzed on an ABI 7300 qRT-PCR machine (Applied Biosystems, U.S.A.).

Quantitative PCR for Col2a1, Col10a1, Pparγ, Mmp13, Sox9, and aggrecan was conducted using transStart Green qPCR SuperMix (Transgen, China) in an ABI7300 qRT-PCR machine. The primers used are described in Table 1.

**Table 1:** Primers used in the qRT-PCR assay

Gene	Primer sequence	Fragment length	Annealing temperature
*Col10a1*	F: 5′-GATCATGGAGCTCACGGAAAA-3′	63 bp	60°C
	R: 5′-CCGTTCGATTCCGCATTG-3′		
*Col2a1*	F: 5′-GGTGGAGCAGCAAGAGCAA-3′	83 bp	60°C
	R: 5′-CGTCGCCGTAGCTGAAGTG-3′		
*Aggrecan*	F: 5′-ACTGAAGGACAGGTTCGAGTG-3′	133 bp	60°C
	R: 5′-CACACCGATAGATCCCAGAGT-3′		
*Pparγ*	F: 5′-ACCACGGTTGATTTCTCCAG-3′	242 bp	60°C
	R: 5′-GCTTTATCCCCACAGACTCG-3′		
*Sox9*	F: 5′-GTGCTGAAGGGCTACGACTGGA-3'	159 bp	64°C
	R: 5′-GTTGTGCAGATGCGGGTACTGG-3′		
*Mmp13*	F:5'-GAGTTGGACTCACTGTTGGTC-3'	216 bp	58°C
	R: GCAAGAGTCACAGGATGGTAG-3'		
*β-actin*	F: 5′-GCTGTGTTGTCCCTGTATGC3′	106 bp	56°C
	R: 5′-GAGCGCGTAACCCTCATAGA3′		
*Rno-miR-27a-3p*	RMIRQP0359GeneCopoeia, China	-	60°C
*Rno-miR-27b-3p*	RMIRQP0361GeneCopoeia, China	-	60°C
*Rat-U6*	RMIRQP9003GeneCopoeia, China	-	60°C

Gene expression was normalized to that of the internal control (β-actin) using the 2^−ΔΔ*C*^_t_ method.

### Histochemistry and immunohistochemistry staining

Articular cartilage from the femurs and tibiae of newborn rats was harvested, skinned, and fixed in 4% paraformaldehyde (Beijing DingGuo Biotechnology, China) for 48 h. The specimens were decalcified in 15% EDTA for 7 days, embedded in paraffin, and sliced into 5-µm thick sections. These sections were stained with Hematoxylin and Eosin to evaluate histological structures and with Safranin O to visualize glycosaminoglycan deposition.

For each immunohistochemistry staining experiment, after deparaffinization, rehydration, and several washes with PBS, sections were treated with the S-100 Protein Detection Kit (Immunohistochemistry) (Fuzhou Maixin Biotechnology Development, China). Sections were incubated with an anti-Pparγ antibody (1:200, Santa Cruz Biotechnology, U.S.A.), stained with 3,3′-diaminobenzindine, and counterstained with Hematoxylin. The integrated optical density (IOD) were semi-quantitated using Imageproplus 6.0 software (Media Cybernetics, U.S.A.).

### *In situ* hybridization

For each hybridization experiment, paraffin sections were prepared from rats at 1, 3, and 7 days. All solutions used in these experiments were treated with diethyl pyrocarbonate to prevent degradation by RNases. Sections were dewaxed in water and covered with 3% H_2_O_2_ for 10 min at room temperature, then washed in distilled water. To expose mRNA nucleic acid fragments, slices were digested with 3% pepsin freshly diluted in citric acid at 37°C for 20 min, then washed with PBS. Each slice was treated with an appropriate amount of prehybridization solution and incubated at 41°C for 4 h. Hybridization was performed using *miR-27b* probes (Boster, China) at 41°C for 12 h, and then the excess probes were washed off in successive steps with saline sodium citrate at different concentrations. Slices were incubated with biotinylated murine anti-digoxin at 25°C for 2 h and washed in PBS. Next, streptavidin–biotin complex was added to the sections, incubated at room temperature for 30 min, and the excess was washed off with PBS. Slices were treated with biotinylated peroxidase at room temperature for 30 min, and then washed in PBS. Sections were stained with 3,3′-Diaminobenzindine and counterstained with Hematoxylin. The IOD was semi-quantitated using Imageproplus 6.0 software (Media Cybernetics, U.S.A.).

### Cell transfection

Exponentially growing chondrocytes were seeded in six-well plates at 2 × 10^5^ cells per well and cultured for 24 h. For overexpression of *miR-27b*, pre-*miR-27b* precursor mimics (Ambion, U.S.A.) were delivered using DharmaFECT 1 (Life Technologies, Germany) following the manufacturer’s protocol. Briefly, mimics (75 nM) were mixed with the transfection agent in Opti-MEM (Invitrogen, U.S.A.) for 20 min, and then added to serum-free medium. As control, two groups of cells were transfected with pre-miRNA precursor NC mimics (Ambion) or treated with transfection reagents alone. The medium was replaced 24 h later with hypertrophy-enhancing medium (DMEM/high glucose, 1% ITS +3, 50 µg/ml ascorbate-2-phosphate, 40 µg/ml l-proline, 1 nM triiodothyronine (Sigma–Aldrich, U.S.A.)). Cells were cultured for either another 24 h and subjected to TRIzol RNA isolation, or for 48 h to conduct Western blot analysis. *miR-27b* expression was analyzed via qRT-PCR as described above. For siRNA-mediated gene knockdown, 50 nM siRNA-Pparγ (GenePharma Co., Ltd, China) was transfected into cells following the manufacturer’s protocol above.

### Luciferase assay

The 3′-UTRs of Pparγ were PCR amplified with DNA oligonucleotides flanked by XhoI and NotI restriction sites. Fragments were cloned downstream of the *Renilla* luciferase gene into the pSICHECK2 vector (Promega) in sense and antisense orientation. Chondrocytes were plated into 96-well plates and cotransfected with the described luciferase reporter constructs and 75 nM pre-*miR-27b* precursor using DharmaFECT 1. Luminescence was measured 48 h after transfection using the Dual-Glo Luciferase Assay System (Promega).

### Western blot analysis

After harvesting, total proteins were extracted from the cultures. Protein content was assessed by the Pierce BCA assay kit (Biyuntian, China). Total protein (30 µg) was loaded on to 10% SDS polyacrylamide electrophoresis gels and transferred on to a PVDF membrane. The membrane was immunoblotted overnight at 4°C with antibodies against Col2a1 (1:1000, Abcam, U.S.A.), Sox9 (1:200, Sigma–Aldrich), Runx2 (1:500, Sigma–Aldrich), Mmp13 (1:500, Abcam), Pparγ (1:200, Santa Cruz Biotechnology), Pparγ2 (1:500, Elabscience), Col10a1 (1:200, Abcam), Gapdh (1:2000, Transgen), and β-actin (1:4000, Santa Cruz Biotechnology). The membrane was then incubated with secondary anti-mouse or anti-goat IgG antibodies (1:2000, Transgen) conjugated with peroxidase for 60 min at room temperature. The signal was detected by chemiluminescence using the ECL-Plus Detection System (Transgen). Protein semi-quantitation was based on three independent experiments. The densitometric intensities of protein bands were semi-quantitated using Bandscan 5.0 (Glyko Biomedical, U.S.A.) software and values were normalized to those of β-actin or Gapdh for each sample.

### Oil Red O staining

After 7 days of culture, chondrocytes were fixed in 4% paraformaldehyde, rinsed with water, and then stained for lipids with freshly prepared Oil Red O dye. The IOD of lipid droplets in monolayer cultures was semi-quantitated using Imageproplus 6.0 software (Media Cybernetics, U.S.A.). Five independent and random fields per sample were counted and averaged per experiment.

### Rosiglitazone treatment

Resting chondrocytes were plated in a monolayer for 24 h, then treated with a concentration gradient of rosiglitazone (0, 0.1, 1, 5, 10, and 20 µg/ml) for another 48 h. Relevant protein expression was detected by Western blot analysis.

### Statistical analysis

Statistical analysis was performed using Student’s *t* test and SPSS version 21 software (IBM, U.S.A.). Values are shown as the mean ± S.D. Significance was set at *P*<0.05.

## Abbreviations

Col2a1: Type II collagen
Col10a1: Type X collagen
DMEM: Dulbecco’s modified Eagle’s medium
HDAC4: Histone deacetylase 4
IOD: Integrated optical density
KO: Knockout
Mmp13: Matrix metalloproteinase 13
NC: Negative control
Ppar: Peroxisome proliferator-activated receptor
PTH: Parathyroid hormone
PTHrP: PTH-related peptide
qRT-PCR: Real-time reverse transcription-polymerase chain reaction
Runx2: Runt-related transcription factor 2
Sox9: Sex-determining Region-box 9
TGF-β: Transforming growth factor β
